# A novel clinical tool to predict cancer‐specific survival in patients with primary pelvic sarcomas: A large population‐based retrospective cohort study

**DOI:** 10.1002/cam4.4998

**Published:** 2022-07-07

**Authors:** Chao Huang, Qiang Su, Zichuan Ding, Weinan Zeng, Zongke Zhou

**Affiliations:** ^1^ Department of Orthopedics West China Hospital of Sichuan University Chengdu China

**Keywords:** cancer‐specific survival, nomogram, pelvis, risk stratification system, sarcoma, SEER

## Abstract

**Background:**

Primary osseous sarcoma of the pelvis is rare and has a particularly sinister outcome. This study aims to identify independent prognostic factors of cancer‐specific survival (CSS) in patients with primary pelvic sarcoma (PS) and develop a nomogram to predict 3‐, 5‐, and 10‐year probability of CSS in these patients.

**Methods:**

The Surveillance, Epidemiology, and End Results (SEER) database was used to identify 416 patients with primary PS, who were divided into two groups: a training cohort and a validation cohort. Univariate and multivariate Cox analyses were used to screen independent prognostic factors in patients with primary PS. Based on these independent prognostic factors, a prognostic nomogram was developed to predict 3‐, 5‐, and 10‐year probability of CSS. The nomogram's predictive performance and clinical value were evaluated using the calibration curve, receiver operating characteristic (ROC) curve, and decision curve analysis (DCA). Finally, a mortality risk stratification system was developed.

**Results:**

Tumor size, tumor stage, histological type, surgery, and chemotherapy were identified as independent prognostic factors for the CSS of primary PS patients. Based on these factors, a nomogram was created to predict the 3‐, 5‐, and 10‐year probability of CSS in these patients. The calibration curve, ROC, and DCA indicated that the nomogram performed well and was appropriate for clinical use, with 3‐, 5‐, and 10‐year areas under ROC curve all higher than 0.800. Furthermore, the nomogram‐based mortality risk stratification system could effectively divide these patients into three risk subgroups.

**Conclusions:**

The nomogram constructed in this study could accurately predict 3‐, 5‐, and 10‐year probability of CSS in patients with primary PS. Clinicians can use the nomogram to categorize these patients into risk subgroups and provide personalized treatment plans.

## Graphical abstract

Four hundred and sixteen patients with primary pelvic sarcomas were collected from the SEER database, consisting of 18 cancer registries and covering 30% of the US population. Tumor size, tumor stage, histological type, surgery, and chemotherapy were identified as independent prognostic factors for the cancer‐specific survival (CSS) of patients with primary pelvic sarcomas. Nomograms for predicting 3‐, 5‐, and 10‐year CSS for patients with primary pelvic sarcomas were constructed based on identified prognostic factors. The mortality risk stratification system based on the nomogram could effectively divide patients with primary pelvic sarcomas into three risk subgroups.

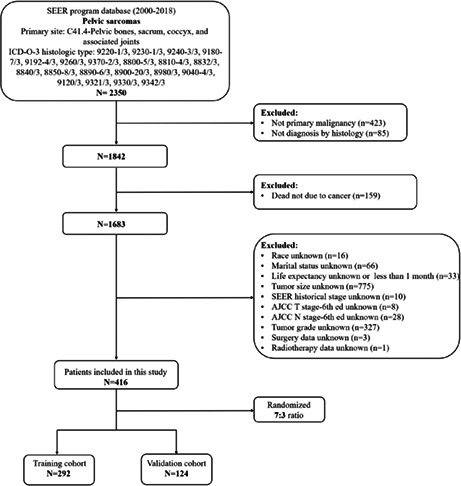

## 1 INTRODUCTION

Primary bone sarcomas are rare bone and joint cancers that account for less than 0.2% of all cancers.^1^ According to the most recent data from the National Cancer Institute's (NCI) Surveillance, Epidemiology, and End Result (SEER) program, the annual rate of new bone sarcoma cases is 1.0 per 100,000, with a mortality rate of 0.5 per 100,000. The 5‐year relative survival rate for bone sarcomas is 66.8%.^2^ In the United States, 3910 new cases of bone sarcoma are expected to be diagnosed in 2022, with approximately 2100 deaths as a result of this cancer, which has become a significant challenge that seriously affects human survival and health.^3^, ^4^

Hu et al. examined all sarcomas of bones and joints diagnosed in the last 40 years and found that patients with sarcomas of the pelvis, sacrum, coccyx, and associated joints had the lowest 5‐year survival rates.^5^ Despite advances in diagnostic imaging, surgical strategies, radiotherapy, neoadjuvant chemotherapy, and targeted therapy that have been applied in the treatment of sarcomas within the last 30 years, the 5‐year survival probability of patients with sarcomas of the pelvis, sacrum, coccyx, and associated joints have not improved significantly. Interestingly, Siracuse et al. found that the suicide rate among patients with primary bone and soft tissue cancer was approximately twice that of the general population in the United States. Patients with pelvic and spine cancer have the highest suicide rate.^6^ Therefore, identifying the factors that limit the benefit of treatment for these patients will thus serve as a valuable reference for developing precision medicine and doctors' treatment plans.

Currently, independent prognostic factors affecting spinal sarcoma survival have been reported, but no comprehensive pelvic sarcoma (PS) studies are available.^7^, ^8^, ^9^, ^10^, ^11^, ^12^, ^13^, ^14^ PS develops slowly, with vague symptoms and rapid growth. It is not easy to diagnose early due to the complex pelvic anatomy and specific location. In practice, when PS is finally diagnosed, it usually has a large tumor volume due to the large and deep pelvic space and the lack of anatomic barriers to tumor diffusion, which often results in compression of adjacent organs, blood vessels, and nerves, making local control difficult.^15^, ^16^ Previous studies have been performed on the survival rates and the related prognostic factors of pelvic osteosarcoma, chondrosarcoma, Ewing sarcoma, and chordoma, but not on PS as a whole.^17^, ^18^, ^19^, ^20^ Although these individual studies provide helpful information for patients with specific PSs, the individual results of these specific tumors limit their application to medical decision‐makers in the overall management of PSs. Therefore, a study of patients with primary PS is required to identify the variables most closely associated with survival to assist clinicians in improving patient counseling and achieving risk‐stratified management and personalized care. Meanwhile, the ultimate goal of cancer treatment and management is to improve patient survival. Overall survival (OS) and cancer‐specific survival (CSS) are frequently used interchangeably, but CSS has a closer relationship to tumor‐mediated patient prognosis than OS and can provide more precise guidance for subsequent analysis of these patients. As a result, we chose CSS as the primary endpoint of the study to identify relevant independent prognostic factors of CSS in patients with primary PS by assessing related details from SEER database to develop a nomogram and risk stratification system for predicting 3‐, 5‐, and 10‐year CSS probability and enabling stratified management of patients with different mortality risks.

## 2 MATERIALS AND METHODS

### 2.1 Database

Since January 1, 1973, NCI's SEER program has collected demographic, neoplastic, and survival data of cancer patients from 18 population‐based cancer registries that cover approximately 30% of the population in the United States (https://seer.cancer.gov/seerstat/, accessed on January 20, 2022).^21^

We downloaded data of patients with primary PS using SEER Stat 8.3.9.2 software with the reference number 16336‐Nov2020 [Incidence‐SEER Research Plus Data, 18 Registries, Nov 2020 Sub (2000–2018)]. This study adhered to (Strengthening the Reporting of Observational Studies in Epidemiology standards [STROBE]).^22^

### 2.2 Population selection

**FIGURE 1:**
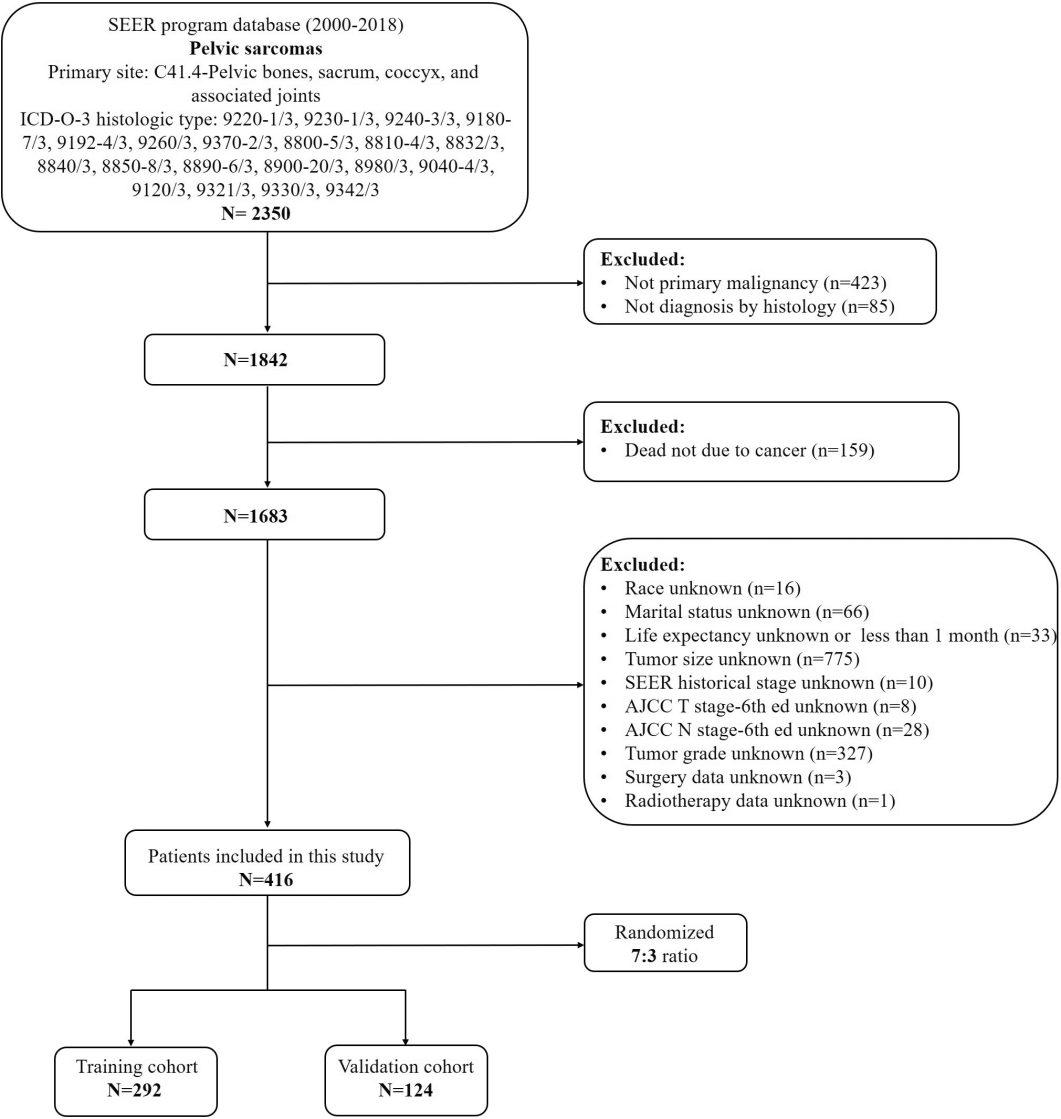
The flowchart of patient selection in this study.

The following were the inclusion criteria: (1) Bones and joints were the site recode ICD‐O‐3/WHO 2008; (2) diagnostic confirmation by positive histology; (3) the primary site of the tumor was the pelvis (C41.4‐pelvic bones, sacrum, coccyx, and associated joints); (4) the designated histologic ICD‐O‐3 codes identified sarcomas for chondrosarcoma (9220–1/3, 9230–1/3, and 9240–3/3), osteosarcoma (9180–7/3, 9192–4/3), Ewing sarcoma (9260/3), chordoma (9370–2/3), and others (8800–5/3, 8810–4/3, 8832/3, 8840/3, 8850–8/3, 8890–6/3, 8900–20/3, 8980/3, 9040–4/3, 9120/3, 9321/3, 9330/3, and 9342/3); (5) primary tumor; and (6) complete follow‐up information. The following were the exclusion criteria: (1) Life expectancy less than 1 month; (2) no information about patients' demographics (age, sex, race, survival time, and marital status), tumor features (tumor size, histological type, and SEER historical stage (tumor stage), Derived American Joint Committee on Cancer (AJCC) T/N stage‐6th ed (T/N stage), and tumor grade), and treatment information (surgery, chemotherapy, and radiotherapy). Finally, 416 patients met the study's criteria and were enrolled (Figure 1).

### 2.3 Variable definitions

Patients' demographic data (age, sex, race, survival time, and marital status), tumor characteristics (tumor size, histological type, tumor stage, T stage, N stage, and tumor grade), and treatment information (surgery, chemotherapy, and radiotherapy) were analyzed in this study. According to X‐tile software, the best cut‐off values for age and tumor size were 51 and 64 years old and 78 and 115 mm, respectively (Supplementary File S1).^23^ Therefore, the age and size of the tumors were categorized into three subgroups, < 51, 51–64, and >64 years old, <78, 78–115, and >115 mm, respectively. Sex was divided into male and female, and the races were classified as black, white, and others (American Indian/AK Native, Asian/Pacific Islander). The two marital status categories were married and unmarried. The tumor stage was categorized into three groups: local, regional, and distant, while the tumor grade was divided into four groups: I, II, III, and IV. The T stage was assigned to T1, T2, and T3, while the N stage was assigned to N0 and N1. AYA site recode/WHO 2008 classified histological types into five subgroups: Chondrosarcoma, osteosarcoma, Ewing sarcoma, chordoma, specified (leiomyosarcoma, synovial sarcoma, biphasic, epithelioid sarcoma, and hemangiosarcoma), and others (fibromyxosarcoma, spindle cell sarcoma, giant cell sarcoma, and sarcoma, NOS). Surgery, chemotherapy, and radiotherapy were identified as “Yes” and “No” groups.

### 2.4 Statistical analysis

All statistical analyses and figures were created using Microsoft Excel 2016 (Microsoft Corp.), R (version 4.0.3), and SPSS (version 22.0) software. A *p*‐value of less than 0.05 was considered statistically significant. At a 7:3 ratio, R software randomly assigned 416 patients to a training and validation cohort. The training cohort was used to identify CSS‐related independent prognostic factors and construct a prognostic nomogram, and the performance of the nomogram was validated using the validation cohort. The study's primary endpoint was CSS, determined as the time between diagnosis and death caused solely by this malignancy. First, Kaplan–Meier method and univariate Cox regression analysis identified significant differences between the enrolled variables. The impact of these variables on these patients' CSS was shown using hazard ratios (HR) and 95% confidence intervals (CI). Then, to control for confounding variables, the risk variables (*p* < 0.05) identified by univariate Cox regression analysis were explored further in multivariate Cox regression analysis to identify CSS‐related independent prognostic factors for patients with primary PS. We created a nomogram based on these CSS‐related independent prognostic factors to predict 3‐, 5‐, and 10‐year CSS probability in patients with primary PS. Meanwhile, this nomogram assigned a point to each CSS‐related independent prognostic factor (Supplementary File S2). The nomogram's predictive ability and clinical utility were validated using a calibration curve, receiver operating characteristic (ROC) curve, and decision curve analysis (DCA). Furthermore, we plotted ROC curves for all independent prognostic factors to confirm that nomogram's predictive validity outperformed a single independent prognostic factor. Moreover, the patient's mortality risk score was calculated by adding the points from each CSS‐related independent prognostic factor. The best cut‐off values for the mortality risk score were determined by using X‐tile software. Then, a mortality risk stratification system was developed to classify the mortality risk of these patients into low‐, middle‐, and high‐risk subgroups. Finally, Kaplan–Meier method was used to compute the difference between the three subgroups.

## 3 RESULTS

### 3.1 Patient characteristics

Based on inclusion and exclusion criteria, this study enrolled 416 individuals with primary PS from SEER database between 2000 and 2015. These patients were divided into two groups: a training cohort (292, 70%) and a validation cohort (124, 30%). All patients' demographic and clinicopathologic features are summarized in Table 1. Of these patients, 249 (59.85%) patients were under 51 years, and 143 (34.38%) had tumor sizes between 78 and 115 mm. The major sex and race of the patients were male (249, 59.86%) and white (363, 87.26%), respectively. The disparity in marital status was not apparent. Chondrosarcoma (229, 55.05%), osteosarcoma (88, 21.15%), and Ewing sarcoma (53, 12.74%) were the top three histological types. A total of 136 (32.69%) patients were in the localized stage, 199 (47.84%) patients were in the regional stage, and 81 (19.47%) patients were in the distant stage. The patients' major T and N stages were T2 (263, 63.22%) and N0 (395, 94.95%). For the treatment, 313 (75.24%), 105 (25.24%), and (173, 41.59%) patients received surgery, radiotherapy, and chemotherapy, respectively.

**TABLE 1:** The baseline demographic and clinicopathologic characteristics of the CSS‐related variables of primary pelvic sarcomas

Variables	Training cohort		Validation cohort		Total	
	292	70.00%	124	30.00%	416	100.00%
Age (years old)						
<51	172	58.90%	77	62.10%	249	59.85%
51–64	71	24.32%	30	24.19%	101	24.28%
>64	49	16.78%	17	13.71%	66	15.87%
Sex						
Male	175	59.93%	74	59.68%	249	59.86%
Female	117	40.07%	50	40.32%	167	40.14%
Race						
Black	19	6.51%	9	7.26%	28	6.73%
White	256	87.67%	107	86.29%	363	87.26%
Other	17	5.82%	8	6.45%	25	6.01%
Marital status						
Unmarried	147	50.34%	68	54.84%	215	51.68%
Married	145	49.66%	56	45.16%	201	48.32%
Tumor size (mm)						
<78	88	30.14%	45	36.29%	133	31.97%
78–115	102	34.93%	41	33.06%	143	34.38%
>115	102	34.93%	38	30.65%	140	33.65%
Histological type						
Osteosarcoma	64	21.92%	24	19.35%	88	21.15%
Chondrosarcoma	157	53.77%	72	58.06%	229	55.05%
Ewing sarcoma	41	14.04%	12	9.68%	53	12.74%
Chordoma	15	5.14%	5	4.03%	20	4.81%
Specified	6	2.05%	5	4.03%	11	2.64%
Others	9	3.08%	6	4.84%	15	3.61%
Tumor grade						
Grade I	53	18.15%	30	24.19%	83	19.95%
Grade II	81	27.74%	37	29.84%	118	28.37%
Grade III	61	20.89%	28	22.58%	89	21.39%
Grade IV	97	33.22%	29	23.39%	126	30.29%
Tumor stage						
Localized	93	31.85%	43	34.68%	136	32.69%
Regional	145	49.66%	54	43.55%	199	47.84%
Distant	54	18.49%	27	21.77%	81	19.47%
Derived AJCC T stage						
T1	98	33.56%	49	39.52%	147	35.34%
T2	191	65.41%	72	58.06%	263	63.22%
T3	3	1.03%	3	2.42%	6	1.44%
Derived AJCC N stage						
N0	277	94.86%	118	95.16%	395	94.95%
N1	15	5.14%	6	4.84%	21	5.05%
Surgery						
No	78	26.71%	25	20.16%	103	24.76%
Yes	214	73.29%	99	79.84%	313	75.24%
Radiotherapy						
No	218	74.66%	93	75.00%	311	74.76%
Yes	74	25.34%	31	25.00%	105	25.24%
Chemotherapy						
No	164	56.16%	79	63.71%	243	58.41%
Yes	128	43.84%	45	36.29%	173	41.59%

Abbreviation: CSS, cancer‐specific survival.

### 3.2 Identification of the independent prognostic factors

**FIGURE 2:**
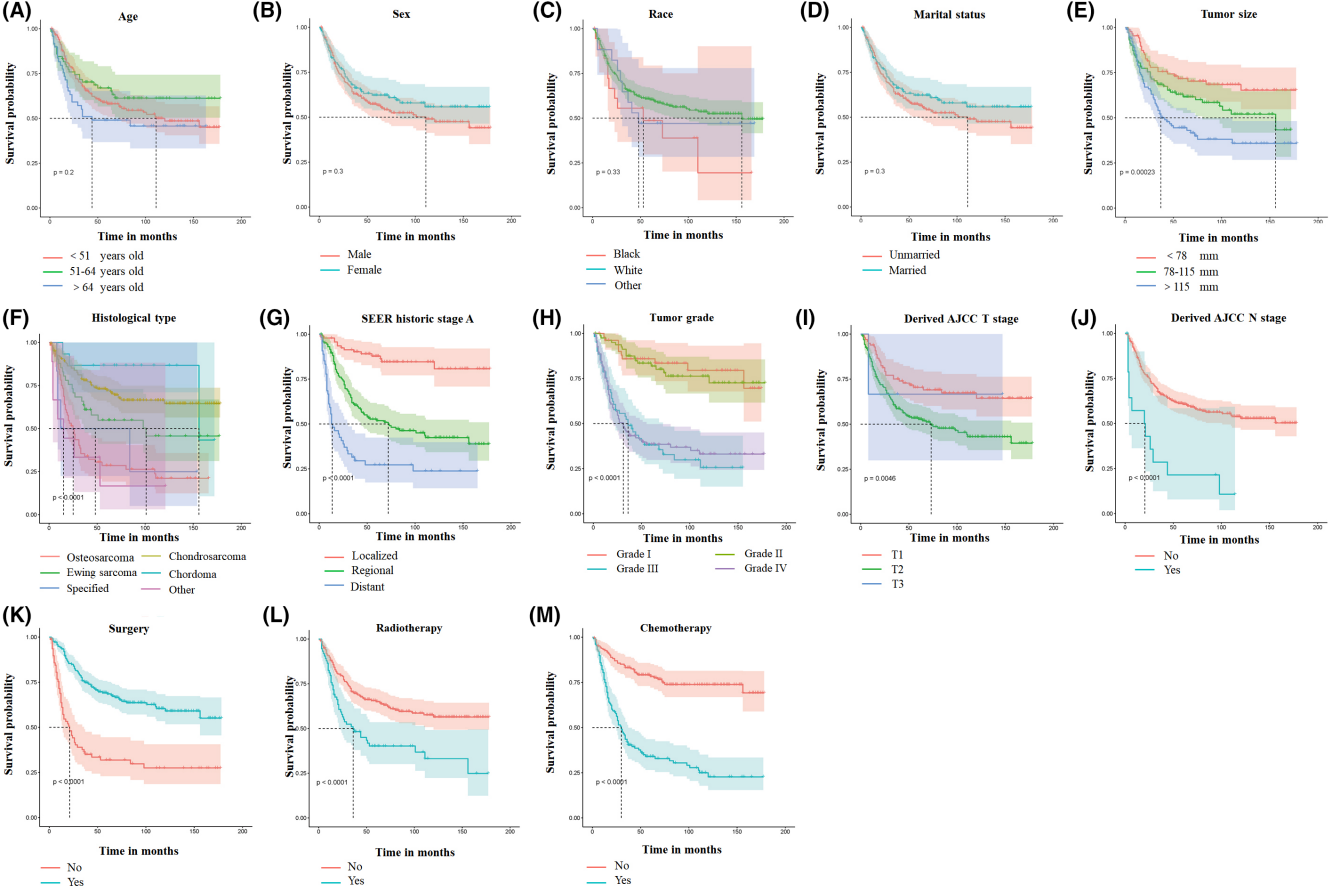
Kaplan–Meier method was performed on cancer‐specific survival (CSS)‐related variables of primary pelvic sarcomas. (A) Age, (B) sex, (C) race, (D) marital status, (E) tumor size, (F) histological type, (G) tumor stage, (H) tumor grade, (I) T stage, (J) N stage, (K) surgery, (L) radiotherapy, and (M) chemotherapy.

Tumor size, histological type, tumor grade, tumor stage, T stage, N stage, surgery, radiotherapy, and chemotherapy were identified as risk‐related variables for CSS using Kaplan–Meier method and univariate Cox regression analysis (*p* < 0.05, Figure 2). They were then investigated using multivariate Cox regression analysis. As shown in Table 2, tumor size, histological type, tumor stage, surgery, and chemotherapy were all found to be independent prognostic factors for CSS in patients with primary PS.

**TABLE 2:** The univariate and multivariate Cox regression analyses of the CSS‐related variables of primary pelvic sarcomas

Variables	Univariate Cox analysis		Multivariate Cox analysis	
	HR (95% CI)	*p*‐value	HR (95% CI)	*p*‐value
Age (years old)				
<51	Reference			
51–64	0.807 (0.518–1.258)	0.344		
>64	1.321 (0.848–2.058)	0.219		
Sex				
Male	Reference			
Female	0.828 (0.579–1.183)	0.300		
Race				
Black	Reference			
White	0.639 (0.343–1.187)	0.156		
Other	0.756 (0.313–1.825)	0.534		
Marital status				
Unmarried	Reference			
Married	0.963 (0.684–1.357)	0.829		
Tumor size (mm)				
<78	Reference		Reference	
78–115	1.584 (0.981–2.558)	0.060	1.511 (0.927–2.462)	0.097
>115	2.464 (1.562–3.886)	<0.001	1.980 (1.215–3.225)	0.006
Histological type				
Osteosarcoma	Reference		Reference	
Chondrosarcoma	0.280 (0.187–0.418)	<0.001	0.838 (0.503–1.395)	0.496
Ewing sarcoma	0.491 (0.291–0.829)	0.008	0.261 (0.148–0.460)	<0.001
Chordoma	0.159 (0.049–0.510)	0.002	0.507 (0.150–1.711)	0.273
Specified	1.016 (0.366–2.821)	0.976	1.181 (0.408–3.419)	0.759
Others	1.308 (0.591–2.892)	0.507	1.437 (0.608–3.397)	0.409
Tumor grade				
Grade I	Reference			
Grade II	1.152 (0.532–2.497)	0.719		
Grade III	5.351 (2.673–10.715)	< 0.001		
Grade IV	5.038 (2.579–9.843)	< 0.001		
Tumor stage				
Localized	Reference		Reference	
Regional	4.356 (2.463–7.703)	<0.001	2.708 (1.493–4.911)	0.001
Distant	9.810 (5.320–18.090)	<0.001	5.183 (2.628–10.222)	<0.001
Derived AJCC T stage				
T1	Reference			
T2	1.933 (1.291–2.895)	0.001		
T3	1.302 (0.178–9.538)	0.795		
Derived AJCC N stage				
N0	Reference			
N1	3.366 (1.855–6.107)	<0.001		
Surgery				
No	Reference		Reference	
Yes	0.324 (0.228–0.460)	<0.001	0.425 (0.280–0.643)	<0.001
Radiotherapy				
No	Reference			
Yes	2.021 (1.410–2.898)	<0.001		
Chemotherapy				
No	Reference		Reference	
Yes	4.262 (2.929–6.201)	<0.001	2.751 (1.635–4.629)	<0.001

Abbreviations: AJCC, American Joint Committee on Cancer; CI, confidence interval; CSS, cancer specific survival; HR, hazard ratio.

### 3.3 Establishment and verification of the nomogram

**FIGURE 3:**
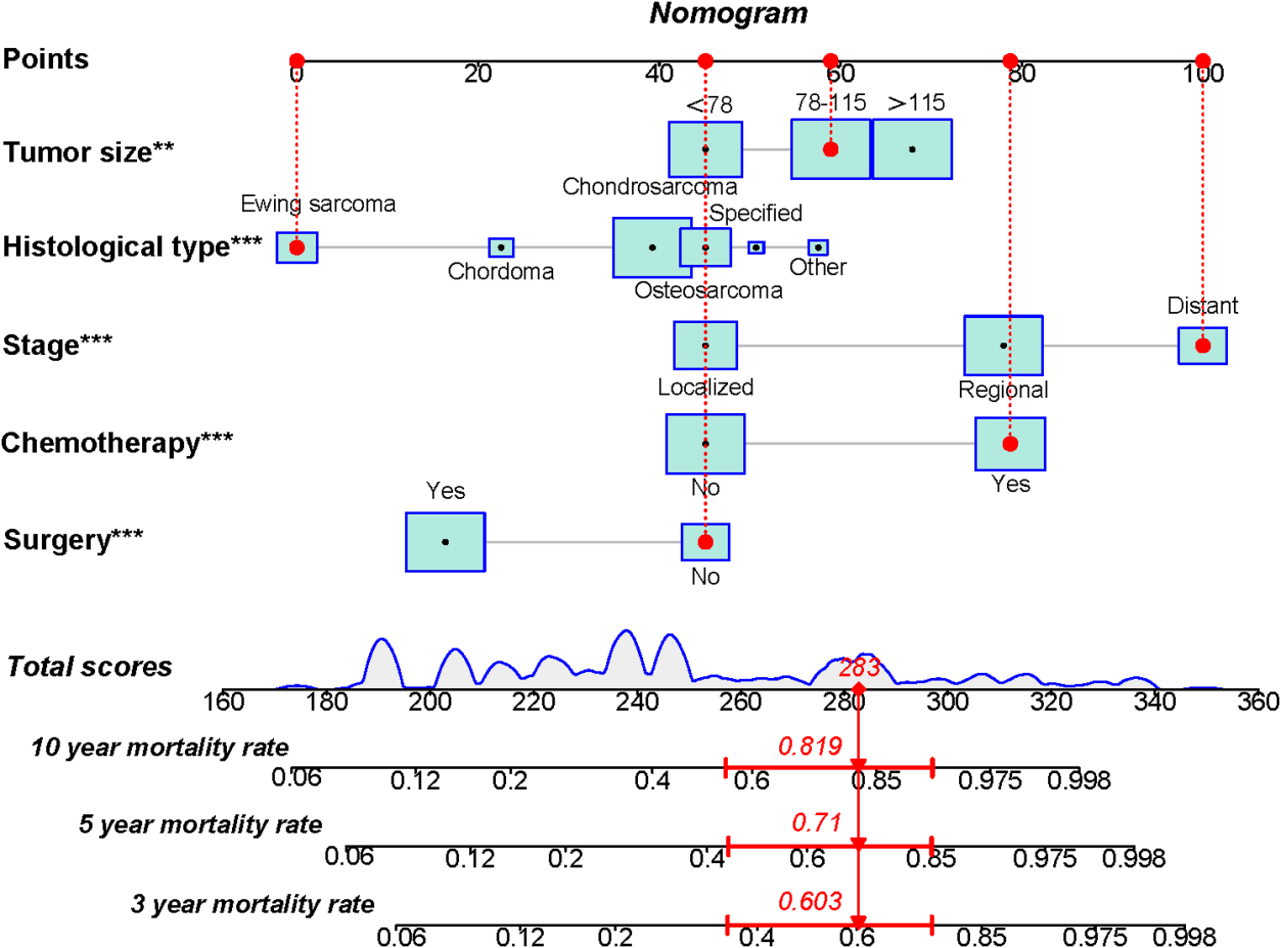
The prognostic nomogram predicts 3‐, 5‐ and 10‐year probability of CSS in patients with primary PS. Specifically, when a patient with primary PS consults about individual survival, we can sum the points of obtained independent prognostic factors to obtain a total score and draw a vertical line from the total score to the bottom timeline to obtain his mortality rate. The probability of survival at the corresponding time can be obtained by subtracting the probability of dead from 1. For example, a patient with a 100 mm diameter primary pelvic Ewing sarcoma with distant stage received chemotherapy without surgical treatment. The corresponding total score of the patient is 59 (100 mm diameter of tumor) + 0 (pelvic Ewing sarcoma) + 100 (distant stage) + 79 (received chemotherapy) + 45 (no surgery) = 283, and the corresponding mortality rate at 3, 5, and 10 years are 0.603, 0.710, and 0.819, respectively, while the patient's corresponding probability of CSS at 3, 5, and 10 years are 0.397, 0.290, and 0.181.

**FIGURE 4:**
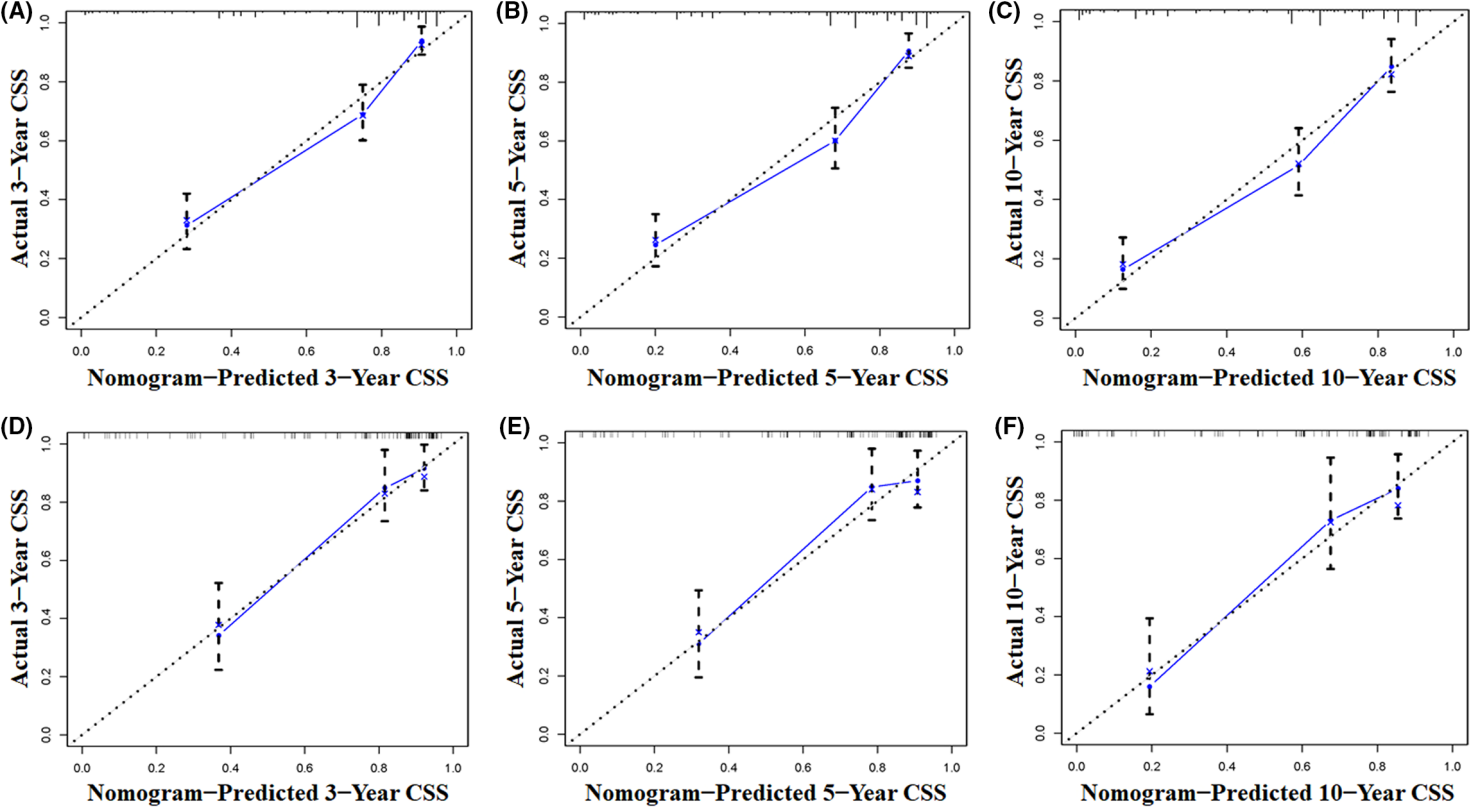
The nomogram's calibration curves at 3‐(A), 5‐(B), and 10‐(C) year in the training cohort and 3‐(D), 5‐(E), and 10‐(F) year in the validation cohort.

**FIGURE 5:**
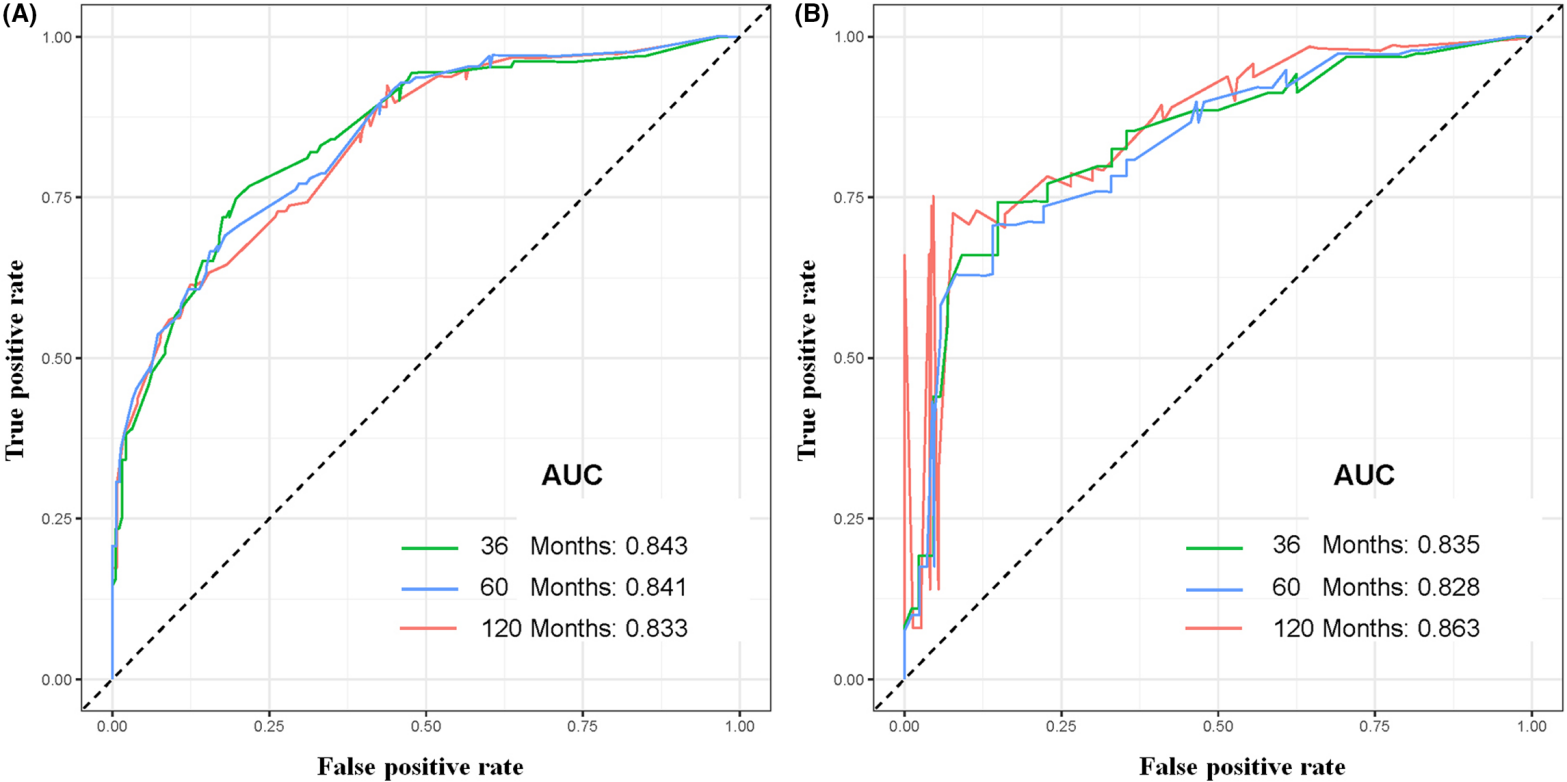
The receiver operating characteristic curves of CSS prediction of patients with primary pelvic sarcomas at 3‐, 5‐, and 10‐year in training (A) and validation (B) cohorts.

**FIGURE 6:**
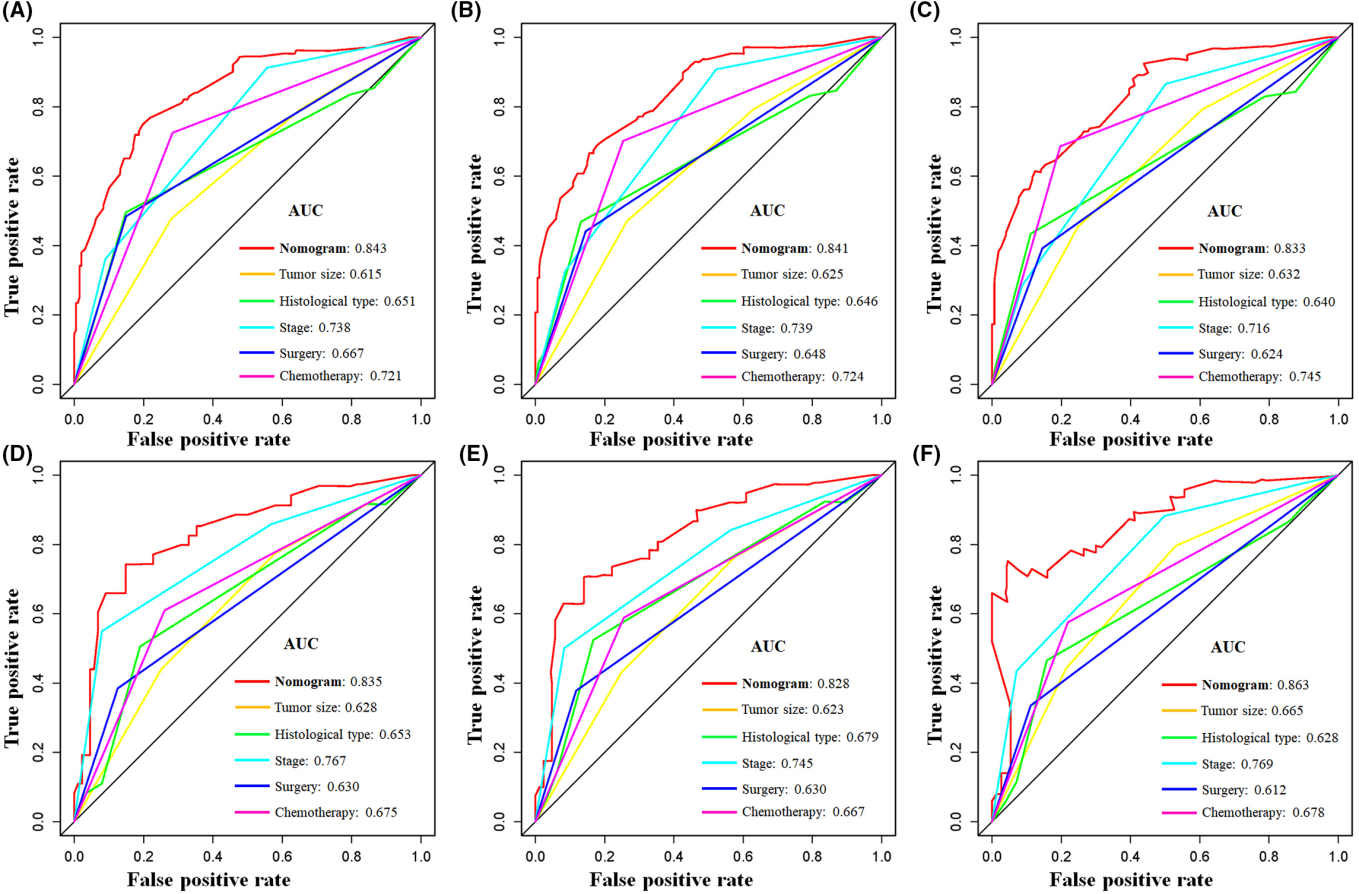
The CSS prediction accuracy was compared using receiver operating characteristic curves between the nomogram and each CSS‐related independent prognostic factor in patients with primary pelvic sarcomas at 3‐(A), 5‐(B), and 10‐(C) years in the training cohort and 3‐(D), 5‐(E), and 10‐(F) years in the validation cohort.

**FIGURE 7:**
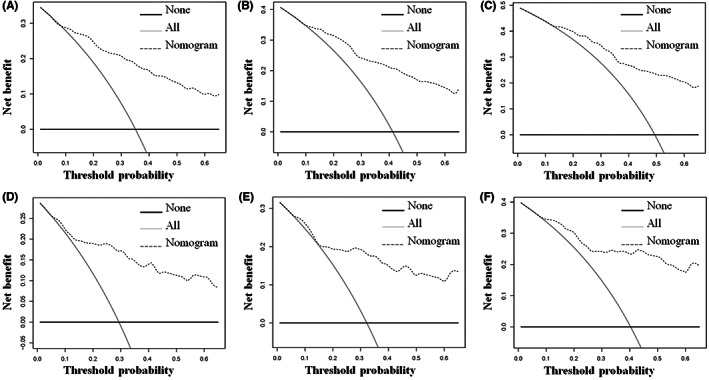
Decision curve analysis was used to predict 3‐(A), 5‐(B), and 10‐(C) year CSS probability in the training cohort and 3‐(D), 5‐(E), and 10‐(F) year probability of CSS in the validation cohort.

Tumor size, histological type, tumor stage, surgery, and chemotherapy were used to develop a prognostic nomogram for predicting 3‐, 5‐, and 10‐year probability of CSS in patients with primary PS using a quantitative method. The points for each independent prognostic factor were summed to obtain the total score for the predicted individual patient (Table 3). A vertical line drawn from the total score row to the bottom timeline can be used to compute the patient's 3‐, 5‐, and 10‐year mortality rate, and the corresponding CSS can be obtained (Figure 3). As revealed in Figure 4, 3‐, 5‐, and 10‐year calibration curves in both cohorts were close to the ideal curve of 45°, demonstrating the nomogram's high prediction accuracy. In the training cohort, 3‐, 5‐, and 10‐year AUC values of ROC curve were 0.843, 0.841, and 0.833, respectively, while those in the validation cohort were 0.835, 0.828, and 0.863, indicating that the nomogram has strong discriminatory power (Figure 5). Meanwhile, as revealed in Figure 6, 3‐, 5‐, and 10‐year AUC values of each independent prognostic factor were lower than those of the nomogram in both cohorts, indicating that the nomogram's prediction accuracy was better than that of every independent prognostic factor. Finally, DCA revealed that the nomogram had an excellent positive net clinical benefit within a specific threshold range, demonstrating the good clinical utility of nomogram (Figure 7).

**TABLE 3:** The detailed point of each independent prognostic factors in the CSS nomogram

CSS‐related independent variables	Corresponding point assignments in CSS nomogram
Tumor size (mm)	
<78	45
78–115	59
>115	68
Histological type	
Osteosarcoma	45
Chondrosarcoma	39
Ewing sarcoma	0
Chordoma	23
Specified	51
Others	58
Tumor stage	
Localized	45
Regional	78
Distant	100
Surgery	
No	45
Yes	16
Chemotherapy	
No	45
Yes	79

Abbreviation: CSS, cancer specific survival.

### 3.4 Development of risk stratification system

**FIGURE 8:**
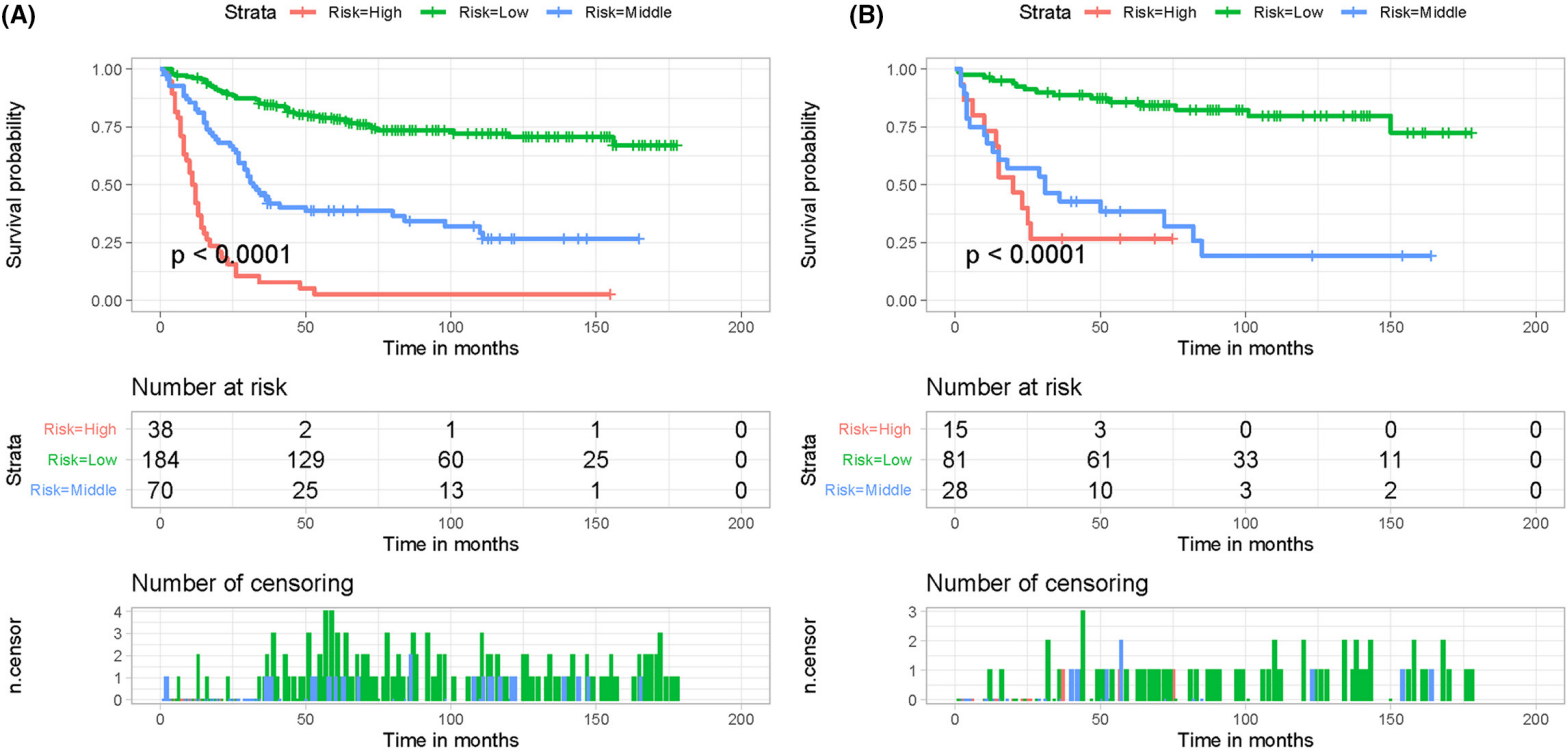
Kaplan–Meier survival analyses were performed to compare CSS in three risk subgroups of patients with primary pelvic sarcomas in training (A) and validation cohorts (B).

A unique mortality risk stratification system was developed for better cancer patient management. Specifically, patients' total scores were calculated based on the nomogram by summing the specific point of the five independent prognostic factors of the patient (Table 3). According to X‐tile software, the best cut‐off values for the total score were 256 and 298 (Supplementary File S1). Thus, patients were further divided into three subgroups based on their mortality risk: low (<256), middle (256–298), and high (>298) groups, and Kaplan–Meier survival curves revealed a significant difference between these three subgroups (*p* < 0.05, Figure 8).

## 4 DISCUSSION

Primary bone sarcoma of the pelvis is a rare bone and joint cancer usually with a particularly sinister outcome. This study aimed to identify independent prognostic factors associated with PS‐specific survival and develop a nomogram for predicting 3‐, 5‐, and 10‐year probability of CSS in these patients. The nomogram, a visual statistical prediction tool for identifying clinical disease‐related prognostic variables, is widely used in cancer management.^24^, ^25^, ^26^, ^27^ Compared to Enneking, AJCC, and TNM staging systems, nomograms can predict and quantify individual patient survival by combining and integrating critical characteristics to improve clinical decision‐making.^28^

This study displayed that tumor size, histological type, tumor stage, surgery, and chemotherapy were independent prognostic factors for 3‐, 5‐ and 10‐year CSS probability in patients with primary PS. The nomogram exhibits a high clinical practice value since it exhibited good agreement between predicted and actual observations. Furthermore, with the increasing application of personalized diagnosis and treatment in cancer management, there is an urgent need to realize personalized management for patients with primary PS to avoid undertreatment for individuals with high mortality risks or overtreatment for individuals with low mortality risks or limited expected benefit from treatment, without wasting healthcare resources or imposing an excessive financial burden on patients. Therefore, based on the constructed nomogram, we developed a mortality risk stratification system for patients with primary PS, which could effectively divide these patients into three risk subgroups (high, middle, and low), formulating treatment strategies adapted to the risk of death, enabling clinicians to achieve patient risk stratification management and targeted treatment to maximize patient benefit.

Tumor size is an independent prognostic predictor of CSS in patients with primary PS. The larger the tumor, the worse the patient's prognosis. The larger the tumor, the more likely it is to compress the visceral and neurovascular bundles surrounding the tumor in the pelvis, causing more obvious signs and symptoms, thus affecting the patient's quality of life. Furthermore, a large tumor size within the complex pelvic structures would cause significant challenges for surgeons attempting to achieve adequate or negative margins during surgical resection. The probability of tumor recurrence and metastasis will also increase, which reduces the survival time of patients.^9^ In addition, tumor stage was an independent prognostic factor in this study, with survival rates for PS patients with local or regional stages present at diagnosis being higher than those for patients with distant stages, demonstrating the importance of early PS diagnosis. However, sarcomas of the pelvis have an insidious onset, and the early symptoms are not always obvious, leading to a delayed diagnosis.^16^ Thus, it remains a challenge to achieve early diagnosis. Moreover, our study disclosed that among sarcomas located in the pelvis, Ewing sarcoma and chordoma had better prognosis in terms of histologic subtypes, followed by chondrosarcoma and osteosarcoma, and the worst were malignant fibromyxosarcoma, spindle cell sarcoma, and giant cell sarcoma, which accounted for 1%–5% of all primary malignant bone tumors.^29^ As a result, when encountering a rare and unknown PS for the first diagnosis in clinical practice, vigilance should be exercised to avoid misdiagnosis and delays in treatment for these patients.

Surgery, chemotherapy, and radiotherapy are currently used to treat primary PS. In the current study, the multivariate Cox regression analysis revealed that surgery and chemotherapy were independent predictive variables for CSS in individuals with primary PS. Patients who received chemotherapy had a significantly lower survival rate than those who did not. Although neoadjuvant chemotherapy combined with surgery is considered the standard of care for osteosarcoma, it should be noted that chemotherapy does not work for all patients with osteosarcoma. Chemotherapy was previously ineffective in elderly patients and those with pelvic osteosarcoma.^5^, ^30^ Marina et al. showed that adding ifosfamide and etoposide to postoperative chemotherapy for osteosarcoma patients who did not respond well to chemotherapy did not translate into a survival benefit but rather increased drug‐related toxicity.^31^ In addition, chondrosarcoma and chordoma are reported to be resistant to chemotherapy. When these patients received chemotherapy, the risks of treatment outweigh the benefits, thus affecting the survival of patients.^12^

Surgery is the most preferred option for PS treatment. In our study, 313 (75.24%) patients received surgery. Surgical resection of the tumor was significantly associated with CSS in univariate and multivariate Cox regression analyses (*p* < 0.001, Table 2), associated with better outcomes for patients with primary PS. The goal of surgical resection should be tailored to the patient's needs to obtain a wide resection and R0 resection margin, thereby reducing the risk of local recurrence or distant metastases. However, the pelvis has a complex anatomy, in which tumors frequently invade adjacent tissues like surrounding viscera (genitourinary and digestive systems), nerves, and blood vessels, making obtaining adequate margins for tumor resection difficult, resulting in a postoperative recurrence rate of 28%–35% for PS, with complications such as infection, bleeding, and shock.^32^ Notably, as treatment techniques advance, the benefits for postoperative PS patients are increased. Using an abdominal aortic balloon occlusion in the excision of pelvic and sacral tumors has shown a significant role in controlling intraoperative bleeding, reducing surgical complications, and improving surgical effectiveness.^15^, ^33^, ^34^ Evrard et al. used patient‐specific instruments (PSI) to achieve surgical resection margins for primary PS and achieved a 0% postoperative tumor recurrence, significantly improving patient outcomes.^32^ Therefore, for PS, surgical resection should be given in time after diagnosis.

Although the nomogram for predicting CSS in patients with primary PS performed well and was reliable, this study had some limitations. First, in this retrospective study, selection bias was unavoidable. Second, SEER database does not include detailed information about surgery, radiotherapy, and chemotherapy, such as specific surgical plans, radiation doses, and chemotherapy regimens, which could be independent prognostic variables for these patients. Third, since the training and validation cohorts in this study originated from the same SEER database, the performance and dependability of the nomogram must be confirmed in other databases.

## 5 CONCLUSION

In patients with primary PS, tumor size, tumor stage, histological type, surgery, and chemotherapy were identified as independent prognostic factors for CSS. These independent prognostic factors were incorporated to construct a prognostic nomogram to predict 3‐, 5‐, and 10‐ year probability of CSS in these patients. Clinicians can use this nomogram to obtain personalized survival possibilities, classify these patients into different mortality risk groups, and provide personalized treatment plans.

## AUTHORS' CONTRIBUTIONS

Chao Huang and Qiang Su designed the study, performed the literature review, extracted the data, and analyzed the pooled data. Zichuan Ding drew the figures and organized the tables. Weinan Zeng and Zongke Zhou provided critical comments and revised the manuscript. All authors read and approved the final manuscript. Chao Huang and Qiang Su contributed equally to this work.

## FUNDING INFORMATION

This research was funded by the Regional Innovation & Cooperation Program of Science & Technology Department of Sichuan Province (grant number: 2021YFQ0028), and the 1·3·5 Project for Disciplines of Excellence, West China Hospital, Sichuan University (grant number: ZYJC18039).

### ETHICAL APPROVAL AND CONSENT TO PARTICIPATE

All methods were carried out in accordance with relevant guidelines and regulations. Data extraction and usage has been approved by SEER Program. All the data can be found in the SEER dataset: https://seer.cancer.gov/seerstat/. We obtained access to the SEER database after obtaining permission to access research data files with the reference number 16336‐Nov2020.

### CONSENT FOR PUBLICATION

All data collected in this study have consent for publication.

## CONFLICT OF INTEREST

The authors declare no competing interests.

## Supporting information


File S1

File S2

## Data Availability

The dataset from the SEER database that was generated and/or analyzed during the current study is available in the SEER dataset repository (https://seer.cancer.gov/). The datasets generated during and/or analyzed during the current study are available from the corresponding author on reasonable request.
