# Supplementary material for: A novel clinical tool to predict cancer‐specific survival in patients with primary pelvic sarcomas: A large population‐based retrospective cohort study
Source: Cancer Med. 2022 Jul 7;12(2):1279–92. doi: 10.1002/cam4.4998 (PMC9883545; doi:10.1002/cam4.4998)
Supplement: Supplementary file 1 — File S1 [file CAM4-12-1279-s001.docx]

**Supplementary Figures**


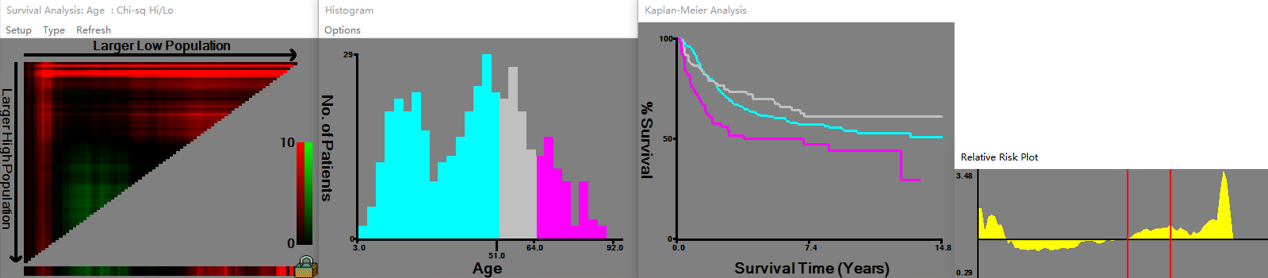


**Supplementary figure 1:** According to X-tile software, the best cut-off values for age were determined to be 51 and 64 (years old).


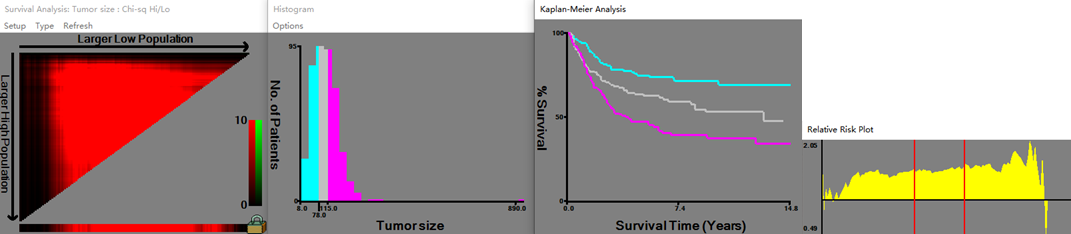


**Supplementary figure 2:** According to X-tile software, the best cut-off values for tumor size were determined to be 78 and 115 (mm).


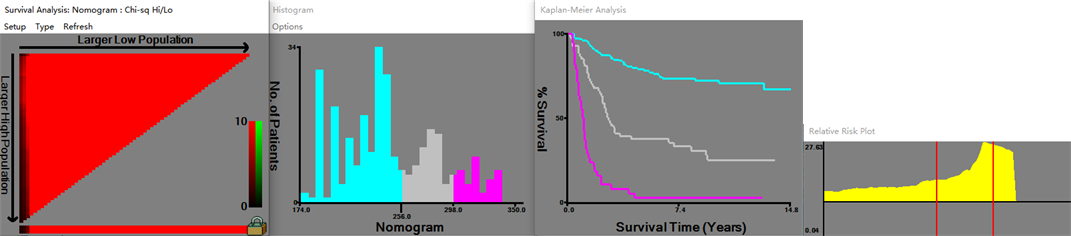


**Supplementary figure 3:** According to X-tile software, the best cut-off values for total score were determined to be 256 and 298.
