# Supplementary material for: A novel clinical tool to predict cancer‐specific survival in patients with primary pelvic sarcomas: A large population‐based retrospective cohort study
Source: Cancer Med. 2022 Jul 7;12(2):1279–92. doi: 10.1002/cam4.4998 (PMC9883545; doi:10.1002/cam4.4998)
Supplement: Supplementary file 2 — File S2 [file CAM4-12-1279-s002.docx]

**Table 3.** The detailed point of each independent prognostic factors in the CSS nomogram.

| **CSS-related independent variables** | **Corresponding point assignments in CSS nomogram** |
| --- | --- |
| **Tumor size (mm)** | |
| ＜78 | 45 |
| 78-115 | 59 |
| ＞115 | 68 |
| **Histological type** | |
| Osteosarcoma | 45 |
| Chondrosarcoma | 39 |
| Ewing sarcoma | 0 |
| Chordoma | 23 |
| Specified | 51 |
| Others | 58 |
| **Tumor stage** | |
| Localized | 45 |
| Regional | 78 |
| Distant | 100 |
| **Surgery** | |
| No | 45 |
| Yes | 16 |
| **Chemotherapy** | |
| No | 45 |
| Yes | 79 |

CSS: cancer specific survival
